# Characterization and epitope mapping of Dengue virus type 1 specific monoclonal antibodies

**DOI:** 10.1186/s12985-017-0856-8

**Published:** 2017-10-02

**Authors:** Wen-Hung Chen, Feng-Pai Chou, Yu-Kuo Wang, Sheng-Cih Huang, Chuan-Hung Cheng, Tung-Kung Wu

**Affiliations:** Department of Biological Science and Technology, National Chiao Tung University, Hsin-Chu, 30068 Taiwan, Republic of China

**Keywords:** Dengue virus (DV), Envelope protein, Monoclonal antibody, Epitope

## Abstract

**Background:**

Dengue virus (DV) infection causes a spectrum of clinical diseases ranging from dengue fever to a life-threatening dengue hemorrhagic fever. Four distinct serotypes (DV1–4), which have similar genome sequences and envelope protein (E protein) antigenic properties, were divided. Among these 4 serotypes, DV1 usually causes predominant infections and fast diagnosis and effective treatments are urgently required to prevent further hospitalization and casualties.

**Methods:**

To develop antibodies specifically targeting and neutralizing DV1, we immunized mice with UV-inactivated DV1 viral particles and recombinant DV1 E protein from residue 1 to 395 (E395), and then generated 12 anti-E monoclonal antibodies (mAbs) as the candidates for a series of characterized assays such as ELISA, dot blot, immunofluorescence assay, western blot, and foci forming analyses.

**Results:**

Among the mAbs, 10 out of 12 showed cross-reactivity to four DV serotypes as well as Japanese encephalitis virus (JEV) in different cross-reactivity patterns. Two particular mAbs, DV1-E1 and DV1-E2, exhibited strong binding specificity and neutralizing activity against DV1 and showed no cross-reactivity to DV2, DV3, DV4 or JEV-infected cells as characterized by ELISA, dot blot, immunofluorescence assay, western blot, and foci forming analyses. Using peptide coated indirect ELISA, we localized the neutralizing determinants of the strongly inhibitory mAbs to a sequence-unique epitope on the later-ridge of domain III of the DV1 E protein, centered near residues T346 and D360 (^346^TQNGRLITANPIVTD^360^). Interestingly, the amino acid sequence of the epitope region is highly conserved among different genotypes of DV1 but diverse from DV2, DV3, DV4 serotypes and other flaviviruses.

**Conclusions:**

Our results showed two selected mAbs DV1-E1 and DV1-E2 can specifically target and significantly neutralize DV1. With further research these two mAbs might be applied in the development of DV1 specific serologic diagnosis and used as a feasible treatment option for DV1 infection. The identification of DV1 mAbs epitope with key residues can also provide vital information for vaccine design.

## Background

Dengue, yellow fever, Japanese encephalitis, tick-borne encephalitis, Zika, and West Nile viruses are all members of the *Flaviviridae* family, a group of viruses notorious for causing human pathogenesis and annually impose severe social and economic burdens on the global society [1]. Among them, dengue virus (DV) infects as many as 100 million people annually and causes an estimated 22,000 deaths per year, with 2.5 billion people living in over 100 countries at risk of exposure [2]. Four distinct serotypes (DV-1, −2, −3, −4), each of which shows some immunological cross-reactivity and differs at the amino acid level of their viral envelope proteins by 25 to 40%, have been identified. A primary infection by DV leads to life-long immunity to this serotype but only partial and temporal immunity to the others [3, 4]. Secondary infection with different serotypes, due to antibody-dependent enhancement phenomena, can progress to a life-threaten dengue hemorrhagic fever (DHF) and dengue shock syndrome (DSS) [5]. In most of the world, DV therapy is still limited to supportive therapy such as judicious fluid management and careful monitoring vital organ function [6]. When proper treatment is not administrated in the early stage of secondary infection with other serotypes, case fatality rates might reach as high as 10–15% in hospitalized patients [2, 4, 7]. In 2015, one DV vaccine CYD-TDV was approved in Brazil, Mexico, and the Philippines, though the efficacy widely varied against all four DV serotypes. CYD-TDV is at approximate 72% and 77% efficacy against DV3 and DV4 separately; however, it is less useful to prevent DV1 and DV2 at 40–50%. [6, 8]. DV1 is usually the predominant serotype in several countries, especially, there were unprecedented outbreaks and caused above 15,000 DV1 cases in southern Taiwan during 2014 and 2015 [9]. More knowledge pools of the immunological differences in sequence and structure between DV1 and other serotypes are needed for the future improvement in the fast screen and emergently providing anti- virus agents.

DV is a spherical lipid bilayer-enveloped virus containing a 10.7-k base positive-sense, non-segmented single-stranded RNA genome [10]. The genome is translated as a single polypeptide, which is then cleaved by viral and cellular proteases into three structural proteins (C, prM/M, E) and seven nonstructural proteins (NS1, NS2A, NS2B, NS3, NS4A, NS4B, NS5) [11, 12]. Among them, the E protein, which plays an important role in viral assembly, receptor attachment, entry and viral fusion, is responsible for eliciting a neutralizing antibody response [13, 14]. The atomic structure of the ectodomain of the E protein has been determined and shown to assemble as dimers with each subunit comprised of three beta-barrel domains [15–18]. Domain I (EDI) consists of a nine-stranded β-barrel with a single *N*-linked glycosylation site in most strains and connects to domain II (EDII) and domain III (EDIII) by four and one linkers, respectively. EDII is a dimerization domain which is composed of two long finger-like structures with a second *N*-linked glycosylation site and a highly conserved 13 amino acid fusion loop. EDIII adopts an immunoglobulin-like fold and putatively contains a cell surface recognition site for receptor interaction [19].

In light of the global clinical and economic burden of dengue infection, both the private and public sector are actively pursuing development of an active vaccine. Identification of a neutralizing epitope may help elucidate virus-host cell interactions and pathogenesis of DHF as well as in significantly reducing morbidity and mortality. Weakly neutralizing, cross-reactive antibodies that bind to virus particles from multiple serotypes, dominantly targeted on prM protein, have been described [20–23]. The E protein represents an interesting target antigen both for diagnosis and neutralization [19, 24–27]. Several serotype-specific neutralizing mAbs against DV1 have been localized to epitopes in EDI and EDIII, however, few have been mapped to specific amino acids or structural determinants [25, 28–33]. In the present study, we identified two neutralizing mAbs that work specifically against the E protein of DV1. The neutralizing epitopes of both mAbs were further identified with synthetic peptides. These findings may be useful for further understanding the mechanism of viral entry and accelerate the development of new vaccines available to areas widely affected by dengue virus.

## Methods

### Chemicals and *E. coli* strain

HAT media supplement (Cat. No. H0262), polyethylene glycol (PEG, Cat. No. P7306) solution, Freund’s adjuvant, and other chemicals were purchased from Sigma-Aldrich (St. Louis, MO, USA). Minimum Essential Medium (MEM), Dulbecco’s Modified Eagle Medium (DMEM), Penicillin/Streptomycin (PS), fetal bovine serum (FBS), HT supplement, and tetramethyl benzidine (TMB) solution were obtained from Invitrogen (Carlsbad, CA, USA). Hybond-C Extra Nitrocellulose (NC) transfer membrane, nProtein A sepharose and Ni-sepharose were purchased from GE healthcare life science (Piscataway, NJ, USA). *Escherichia coli* host strain BL21 (DE3) and the plasmid pET28a were purchased from Novagen (Merck, Darmstadt, Germany). The 96-well microtiter plate used for ELISA was purchased from Nunc (Maxisorp™ surface, no. 442404, Roskilde, Denmark). Horseradish peroxidase (HRP)-conjugated anti-mouse immunoglobulins (IgG) as the secondary antibody used in ELISA, Dot blot, Western blot and focus forming assay (FFA) was purchased from KPL (Cat. No. 074–1806, MD, USA).

### Virus propagation and cell culture

The used prototype of DV strains: DV-1 (Hawaii), DV-2 (*NGC*), DV-3 (*H87*), DV-4 (*H241*), and control Japanese encephalitis virus (JEV *T1P1*) were propagated in *Aedes albopictus* cells (C6/36, ATCC: CRL-1660). The culture supernatants were collected, filtrated with 0.45 μm filter to remove cell debris, aliquoted and frozen at −80 °C until use. The clinical DV strains collected in Taiwan: DV1 (776669A/1988, 766733A/1995, SD9506982/2006), DV2 (766635A/1987, PL046/1995, SD9500852/2006), DV3 (333134A/1994, 19,990,628/1999, H950421/2006), DV4 (866146A/1994, 2 k0713/2000, KSD9301974/2004) were also propagated in C6/36 cells and applied in dot blot analysis and indirect immunofluorescence assays (IFA) experiments. Viruses were tittered by ten-fold serial dilution on Hamster kidney cells (BHK-21, ATCC: CCL-10) followed by overlay medium (MEM containing 2% heat-inactivated FBS, 1% PS and 1% carboxymethyl cellulose). After six-day incubation, plaques were determined by crystal violet staining [34]. C6/36 cells were maintained in MEM supplemented with 1% PS and 10% heat-inactivated FBS at 28 °C with 5% CO_2_. BHK-21 cells and African green monkey kidney cells (Vero, ATCC: CCL-81) were grown in MEM supplemented with 1% PS and 10% heat-inactivated FBS at 37 °C with 5% CO_2_. Myeloma cells (FO, ATCC: CRL-1646) were grown in DMEM supplemented with 0.15% of sodium bicarbonate, 1% PS, and 10% of heat-inactivated FBS at 37 °C with 5% CO_2_. Hybridoma cells were grown in HY medium (DEME supplemented with 0.15% of sodium bicarbonate, 1% PS, 1% HT supplement and 10% of heat-inactivated FBS) at 37 °C with 5% CO_2_.

### Cloning, expression and purification of DV1 E395 protein

Molecular cloning, protein expression, and purification of DV1 E395 (residue 1–395) were performed according to procedures provided in a previous publication [27]. The protein identities of the SDS-PAGE bands corresponding to DV1 E395 were confirmed by matrix-assisted laser desorption/ionization time-of-flight mass spectrometry (MALDI-TOF-MS).

### Generation and identification of mAbs against DV1 E395 protein

The preparation and identification of mAbs against the DV1 E395 protein were performed as previously described with slight modifications [27]. Six-week-old female BALB/c mice were first intraperitoneally immunized with two doses of UV-inactivated DV1 (approximately 10^5^ particles) at two-week intervals, followed by three boosts of recombinant DV1 E395 (100 μg) at two-week intervals. Three days later, all mice were bled and the serum samples were tested by indirect ELISA with DV1 E395 protein (100 μL, 20 μg/mL) as the coating antigen to determine which mouse had the greatest response. This mouse was euthanized and its splenocytes were fused with FO cells to generate hybridoma cell lines according to the previous published procedures [35]. Briefly, the spleen was removed from the immunized mouse and spleen cells were mixed with FO cells at 4:1 ratio. After washed with 10 mL of DMEM for three times, spleen/FO cells were fused with 1 mL of PEG solution and incubated at 37 °C for 1 min with gently stirring. The PEG solution was diluted slowly and gently stirred with 10 mL of DMEM. Subsequently, the cells were centrifuged at 450 × *g* for 5 min and then washed with 10 mL of DMEM for three times. The cell pellet was resuspended with DMEM containing 20% heat-inactivated FBS, 1% PS and 1% HAT media supplement. One hundred microliter of the resuspended fused cells was distributed in 96-well tissue culture plates for antibody screening. On the other hand, the DV1 virus was propagated in *Aedes albopictus* clone C6/36 cells in MEM medium supplemented with 10% fetal calf serum. The culture supernatants were collected, filtrated with 0.45 μm filter to remove cell debris, aliquoted (10^5^ PFU/mL) and frozen at −80 °C until use. The hybridoma cell lines were screened by an indirect ELISA for the presence of mAbs against DV1 E395 and DV1 infected C6/36 cell lysates. The Positive hybridoma cell lines were cloned by limiting dilution to generate mAbs.

### Serotype-specificity and cross-reactivity evaluation of the mAbs

The serotype-specificity and cross-reactivity of the mAbs were evaluated by indirect ELISA, Western blot analysis, Dot blot, indirect immunofluorescence assays (IFA), and focus forming assay (FFA). In parallel, a mAb HB114 (D3-2H2–9-21, ATCC) which reacts with all members of the DV complex was used as a positive control. Cell lysates of the above-mentioned prototype and clinical DV strains-, JEV-, and mock-infected C6/36 cells were used to evaluate the serotype specificity and cross-reactivity.

### Indirect ELISA

To perform indirect ELISA assay, a 96-well microtiter plates were coated with 100 uL of prototype and clinical DV strains (10^5^ PFU/mL), JEV-, or mock-infected C6/36 cell lysates and incubated at room temperature for 1 h. After blocking with 5% skim milk, 100 uL of hybridoma supernatants was added to the plates and incubated at room temperature for 1 h. The plates were washed three times with PBS containing 0.5% skim milk and 0.05% Tween-20. One hundred microliters of horseradish peroxidase (HRP)-conjugated anti-mouse immunoglobin G (1:5000 dilution, Abcam, USA) was added to the plates and incubated at room temperature for 1 h. The plates were washed three times with PBS containing 0.5% skim milk and 0.05% Tween-20 and incubated with 100 uL of TMB solution. The reaction was stopped with 2 M sulfuric acid and the plates were read using a microplate reader at 450 nm.

### Dot blot analysis

To perform dot blot assay, 50 μL (20 μg/mL) of different targets were spotted on Hybound-C NC membrane and detected with SNAP i.d.™ Protein Detection System (Millipore) according to the user’s guide. Nonspecific antibody binding sites were blocked with 0.5% (*w*/*v*) skim milk in PBS containing 0.1% (*v*/v) Tween-20. The membrane was incubated with DV1-E1 (or DV1-E2) and then HRP-conjugated anti-mouse IgG (1:10,000 dilution) for 10 min at room temperature. The signal was developed with Western lighting-ECL kit (PerkinElmer life science) and exposed to Fuji X-ray medical film.

### Western blot analysis

Lysates from DV strains, JEV-, or mock-infected C6/36 cells were mixed with loading buffer (125 mM Tris–HCl pH 6.8, 5% (*v*/v) 2-mercaptoethanol, 5% (*w*/*v*) SDS, 0.05% (v/v) bromophenol blue, and 25% (v/v) glycerol) and boiled for 10 min. After electrophoresis, proteins were transferred to a Hybound-C NC membrane. Following nonspecific blocking with blocking buffer (0.5% (w/v) skim milk in PBS containing 0.1% (v/v) Tween-20), the membrane was incubated with hybridoma supernatant and HRP-conjugated anti-mouse IgG (1:10,000 dilution) and detected with SNAP i.d.™ Protein Detection System, as previously described.

### Indirect immunofluorescence assays

The prototype and clinical DV strains, JEV-, or mock-infected C6/36 cells as previously described were scraped and fixed on slides with cold acetone for 10 min at 4 °C. The slides were individually incubated with DV1-E1, DV1-E2, and HB114 mAbs for 30 min at 37 °C [36]. After washing with PBS and air-dried, FITC-conjugated goat anti-mouse IgG (1:1000 dilution, Kirkegaard & Perry Laboratories, USA) was added and incubated at 37 °C in the dark for 30 min. Similarly, the cells were mounted in anti-fading medium and were observed using fluorescence microscope after washing and air-drying.

### Infectivity titration with focus-forming assay

Virus titers were determined by focus-forming assay (FFA). Vero cells were seeded at 2 × 10^4^ cells per well in 96-well plates and cultured 24 h to form a confluent monolayer. A 10-fold serially diluted virus was added and the wells were incubated at 37 °C for 2 h. Next, the wells were overlaid with 100 μL overlay medium (1% methylcellulose in DMEM containing 1% FBS) and incubated at 37 °C for 3 days. The overlay medium was removed and the wells were washed with PBS, followed by fixation with 4% paraformaldehyde in PBS for 20 min at room temperature. After removal of fixation solution, the cells were permeabilized and stained with DV1-E1 or DV1-E2 for 30 min followed by incubation for 30 min with HRP-conjugated goat anti-mouse IgG (1:500 dilution). The blue focus-forming spots were visualized using peroxidase substrate (0.5 mg/mL 3,3-diaminobenzidine tetrahydrochloride dehydrate containing 0.08% NaCl and 0.01% H_2_O_2_ in PBS). Photographs were taken on the same day as staining.

### Inhibition of DV1 infection in C6/36, Vero, and BHK-21 cells by anti-E mAbs

Different target cells, Vero, C6/36 or BHK-21, were seeded at 1 × 10^4^ cells in 24-well plates for 24 h prior to performing virus infection. One hundred and fifty microliter hybridoma supernatants (10 μg/mL, approximately 1.5 μg total of anti-E mAb) were mixed with 50 μL of DV1 (8 × 10^3^ PFU/mL) and pre-incubated at 37 °C for 1 h before adding into the cultured 24-well plates with target cells. In parallel, HY medium containing mouse IgG was incubated with DV1 as the negative control. The antibody-virus mixture was incubated in triplicate wells with target cells at 37 °C for 1 h, with shaking every 15 min. After the incubation, unbound antibody-virus mixtures were removed and infected cells were overlaid with medium (MEM containing 5% FBS and 1% PS). Plates were incubated in 5% CO_2_ at 37 °C for 2 days. The supernatants were collected and the viral titers were determined by FFA.

### Epitope type determination of anti-E mAbs

To determine the conformational- or sequence-dependent type of epitope, the virus-antibody reactivity was determined by ELISA with and without heated treatment, respectively. One hundred microliters of different serotype DV and JEV-infected C6/36 cell lysates were either heated at 95 °C or retained at 37 °C for 10 min before adding anti-E mAbs. After removal of cell lysates, 5% skim milk in PBS was used as the coating on the dish and the remaining procedures were identical to that of indirect ELISA.

### Epitope mapping by peptide-coated ELISA

Ten 5 mer-overlapped synthetic oligopeptides spanning the EDIII of DV1 E protein (amino acids from 296 to 395) were synthesized as followings: P1: GMSYVMCTGSFKLEK (aa 296–310); P2: FKLEKEVAETQHGTV (aa 306–320); P3: QHGTVLVQVKYEGTD (aa 316–330); P4: YEGTDAPCKIPFSTQ (aa 326–340); P5: PFSTQDEKGVTQNGR (aa 336–350); P6: TQNGRLITANPIVTD (aa 346–360); P7: PIVTDKEKPVNIEAE (aa 356–370); P8: NIEAEPPFGESYIVV (aa 366–380); P9: SYIVVGAGEKALKLS (aa 376–390); P10: ALKLSWFKKG (aa386–395). A 96-well plate was coated with the synthesized peptides (1 μg in 100 μL of PBS) and incubated at room temperature for 1 h. After blocking with 5% skim milk, the plate was added with 100 μL of mAb (10 μg/mL from supernatant) and the remaining procedures were the same as that of indirect ELISA.

### EDIII sequence analysis and protein structure graphic

Amino acid sequence alignment of EDIII from different DV serotypes and JEV was created with the program ClustalW. Protein blast was performed via the NCBI non-redundant protein database using the blastp program. The crystal structure figure was prepared using the atomic coordinates of DV1 EDIII (RCSB accession number 3G7T) with the molecular graphic program *Pymol* (http://www.pymol.org).

## Results

### Generation and evaluation of serotype-specificity of anti-E mAbs

**Fig. 1:**
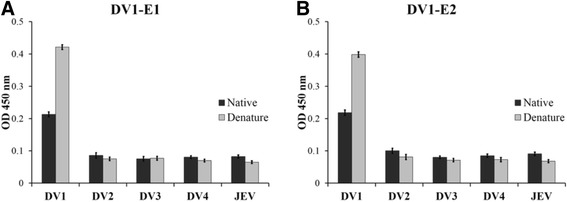
Preliminary determination of serotype specificity of selected anti-E mAbs by indirect ELISA. Four serotypes of DV (DV1, Hawaii; DV2, NGC; DV3, H87; DV4, H241) and JEV (T1P1)-infected C6/36 cell lysates, either native (dark column) or heat-treated (grey column, 95 °C for 10 min) DV1 E395 protein were coated on ELISA plates and reacted with (**a**) DV1-E1 and (**b**) DV1-E2 mAbs, respectively. Error bars indicate standard deviations (SD)

**Fig. 2:**
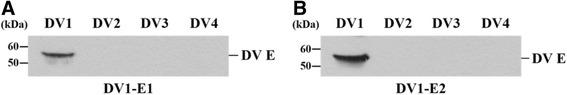
Identification of mAbs against E protein of DVs by western blot analysis. Four serotypes of DV- and JEV-infected C6/36 cell lysates were harvested and fractionated in 10% SDS-polyacrylamide gel. The blots were incubated with (**a**) DV1-E1 and (**b**) DV1-E2, respectively

To develop neutralizing monoclonal antibodies (mAbs), BALB/c mice were immunized with UV-inactivated DV1 viral particles and then boosted with recombinant DV1 E395 protein. The mice were bled and the serum samples were tested by ELISA to determine the sample with the greatest response to recombinant DV1 E395. The mouse with the highest titer was euthanized and its splenocytes were fused with FO cells to generate hybridoma cell lines. A total of 12 hybridoma clones that exhibited strong positive reactivity with both the recombinant DV1 E395 protein and DV1 infected C6/36 cells in ELISA were isolated and cloned. The preliminary serotype specificity of the mAbs was further identified by indirect ELISA. Of the 12 mAbs against to DV1 E protein (anti-E mAb), 2 clones (DV1-E1 and DV1-E2) specifically reacted to DV1 with no cross-reactivity to the other three DV serotypes and JEV in the indirect ELISA (Fig. 1). The remaining 10 mAbs had cross-reactivity to the four DV serotypes and JEV in different cross-reactivity patterns (data not shown). The serotype specificity of these two mAbs was further confirmed by an immunoblotting assay. Consistent results were also obtained with DV1-E1 and DV1-E2 mAbs, demonstrating that mAb DV1-E1 and DV1-E2 are DV1-targeting (Fig. 2).

### Evaluation of serotype-specificity of anti-E mAbs to clinical dengue viruses

**Fig. 3:**
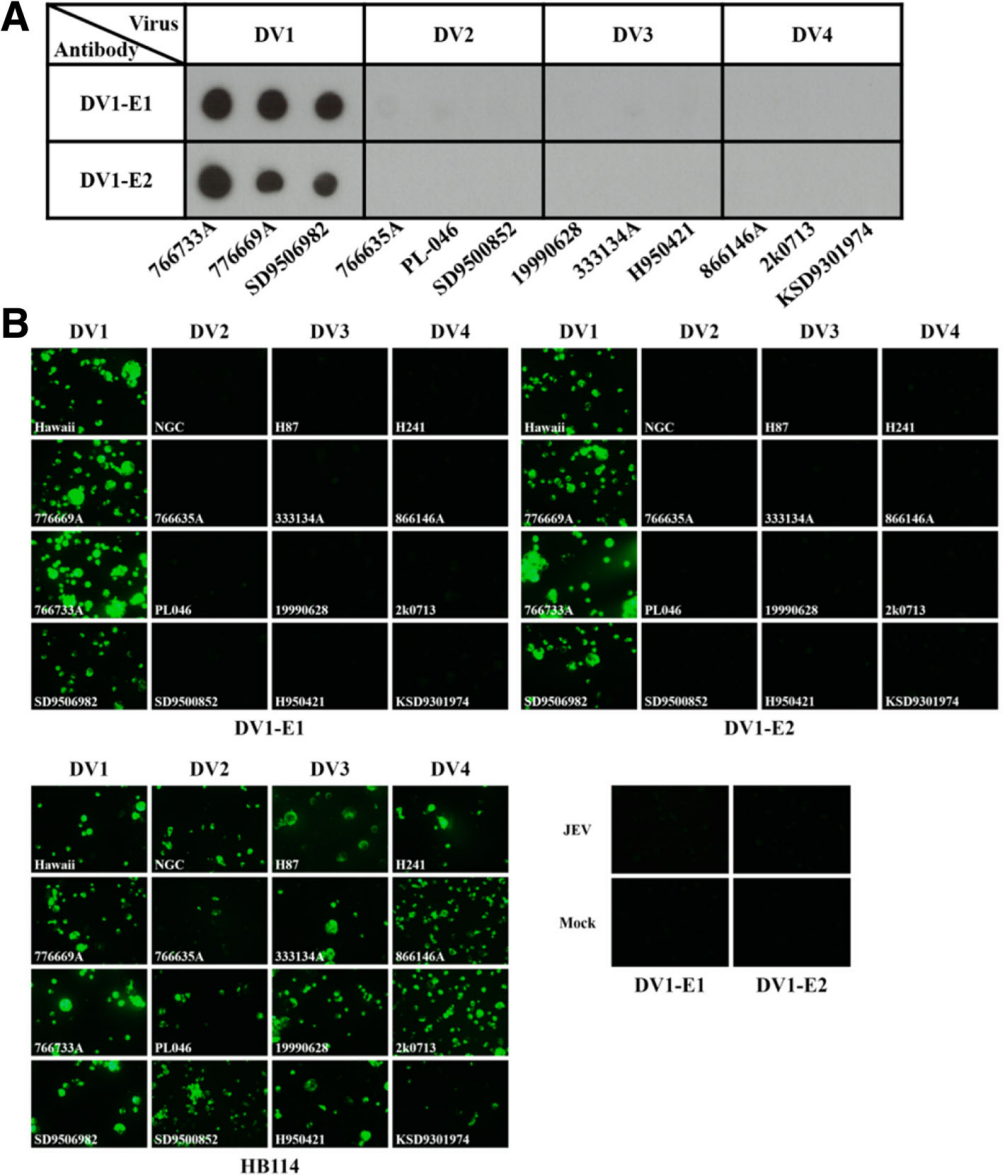
Evaluation of serotype specificity of anti-E mAbs. **a** Dot blot analysis using lysates from C6/36 cells infected with clinical DV strains collected in Taiwan as a target, which were spotted on NC membrane and reacted with DV1-E1 and DV1-E2, respectively. **b** Identification of the serotype specificity of anti-E mAbs by IFA. Prototype or clinical samples collected in Taiwan of DV-infected C6/36 cells were scraped and fixed on slides, then reacted with DV1-E1 and DV1-E2. A monoclonal antibody (HB114) directed against dengue E protein was reacted with the virus-infected cells as a positive control. JEV (NAK strain) or mock-infected C6/36 cells were stained with the anti-E mAbs as negative controls

To further confirm the serotype specificity of the selected two mAbs, clinical DV strains collected in Taiwan between 1987 and 2006, were tested by DV1-E1 and DV1-E2 using dot blot and IFA. In parallel, HB114, a monoclonal antibody which reacts with all members of the dengue virus complex, was used as a positive control. As shown in Fig. 3, both DV1-E1 and DV1-E2 specifically reacted to one DV1 prototype strain (Hawaii) and three DV1 clinical strains (766733A, 776669A, and SD9506982) with no cross-reactivity to prototype and clinical strains of other three DV serotypes in the dot blot and IFA. These results further confirmed the serotype-specificity of the DV1-E1 and DV1-E2.

### Inhibition of DV1 infection by anti-E mAbs

**Fig. 4:**
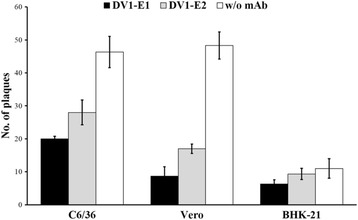
Inhibitory effect of anti-E mAbs on DV1 infection of C6/36, Vero, and BHK-21 cells. C6/36, Vero, and BHK21 cells were infected with DV1 in the absence or presence of DV1-E1 (dark column) or DV1-E2 (gray column). Mouse IgG (w/o mAb; white column) was used as the control for evaluation the inhibitory effects of two anti-E mAbs. After incubation at 37 °C for 48 h, the cells were harvested and the viral titers were determined by FFA. The data are the means and standard deviations from three independent experiments

To characterize the retention of binding versus the neutralizing activity of the generated anti-E mAbs, a semi-quantitatively plaque reduction assay of hybridoma supernatants of both mAbs was performed. DV1 viruses were pre-incubated with DV1-E1 or DV1-E2 and were used to infect C6/36, Vero, and BHK21 cells, respectively. The number of viral foci was determined from three experiments and normalized using a mouse IgG control to calculate the percentage of inhibition. The results showed that DV1-E1 and DV1-E2 were able to inhibit the infection of C6/36 and Vero by DV1 (Fig. 4). In C6/36 and Vero cells, DV1-E1 reduced plaque numbers by 57% and DV1-E2 reduced plaque numbers by 40%. In Vero cells, DV1-E1 and DV1-E2 reduced plaque numbers by 82% and 64%, respectively.

### Epitope localization of anti-E mAbs

**Fig. 5:**
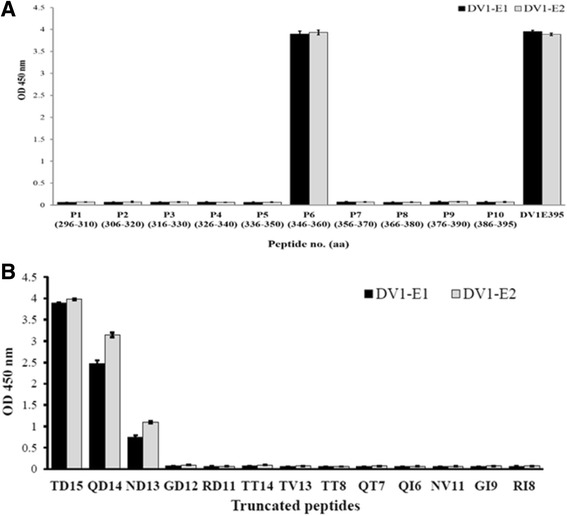
Epitope mapping of generated anti-E mAbs by peptide-coated ELISA. **a** Ten synthesized peptides (P1-P10) spanning the EDIII region were coated on ELISA plates and reacted with DV1-E1 (dark column) and DV1-E2 (gray column). **b** Twelve truncated peptides (listed as in Table 1), which were synthesized based on the peptide P6 (also named TD15), reacted with DV1-E1 (dark column) and DV1-E2 (gray column). Error bars indicate standard deviation

We designed to create anti-E mAbs specifically targeting EDIII region of DV1 E protein. To localize linear epitopes within the EDIII, nine 15-mer and one 10-mer peptides were synthesized and reacted with DV1-E1 and DV1-E2 by ELISA. Only synthesized P6 (^346^TQNGRLITANPIVTD^360^) peptide was recognized by both DV1-E1 and DV1-E2 (OD_450_ = ~4.0), but the other peptides failed to react with these two mAbs (OD_450_ < 0.1). This indicated that the sequence of the epitope recognized by DV1-E1 and DV1-E2 was located in P6. For further epitope determination, trypsin was applied to digest P6 into two fragments: ^346^TQNGR^350^and ^351^LITANPIVTD^360^. The obvious decrease of the OD_450_ signal (from ~4.0 to ~0.5, data not shown) indicated that the epitope spans across the trypsin cleavage site. In order to identify the minimal epitope size, a series of truncated peptides derived from P6 (also named TD15) (Table 1) were synthesized and subjected to ELISA. ELISA results showed that QD14, which removes Thr^346^ from the original P6 sequence, slightly affected its recognition ability by DV1-E1 and DV1-E2 and reduced the binding affinity to 79% and 64% of its original intensity, respectively (Fig. 5b). Alternatively, TT14, which eliminates the Asp^360^ residue located at the *C*-terminus, completely abolished the recognition by DV1-E1 and DV1-E2. Furthermore, binding affinity for ND13 and GD12 was significantly reduced to 28% and 2% for DV1-E1 and to 19% and 2% for DV1-E2, respectively. Other truncated peptides, which omitted additional residues either from the D^360^ or ^346^TQN, could not be recognized by DV1-E1 and DV1-E2, respectively. Together these results reveal that TD15, residue 346–360 of EDIII, is the minimal length required for the reactivity of the linearized epitope to be recognized by DV1-E1 and DV1-E2.

**Table 1:** Amino acid sequence of truncated peptides, which were synthesized based on the P6 peptide

Name	Amino acid sequence
TD15(P6)	T Q N G R L I T A N P I V T D
QD14	Q N G R L I T A N P I V T D
ND13	N G R L I T A N P I V T D
GD12	G R L I T A N P I V T D
RD11	R L I T A N P I V T D
TT14	T Q N G R L I T A N P I V T
TV13	T Q N G R L I T A N P I V
NV11	N G R L I T A N P I V
GI9	G R L I T A N P I
RI8	R L I T A N P I
TT8	T Q N G R L I T
QT7	Q N G R L I T
QI6	Q N G R L I

### Structural properties and sequence alignment of epitope

**Fig. 6:**
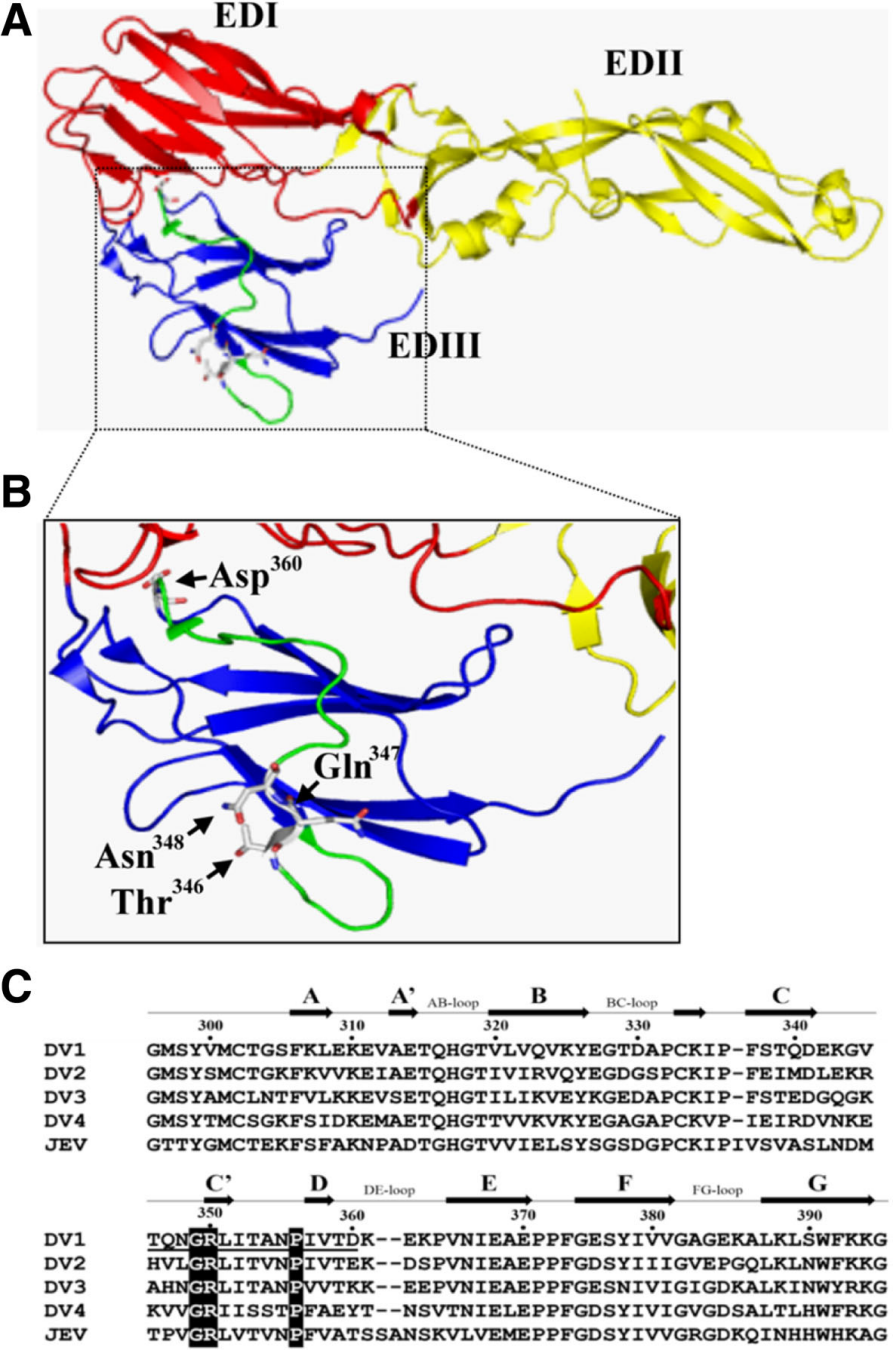
Overview of the DV envelope protein and epitope localization of anti-E mAbs. **a** Crystal structure of the post-fusion DV1-soluble fragment E (sE) (PDB accession number 3G7T), with subunits highlighted and colored by domains (EDI, red; EDII, yellow; EDIII, blue). The location of the mAb epitope (residues 346–360) is highlighted by green. **b** Enlargement of the EDIII region (blue) with mAb epitope region (green). The major residues of the loop of mAb epitope are marked, the starting residue Thr^346^, and three contact residues Gln^347^, Asn^348^, and Asp^360^. **c** Amino acid sequence alignment of four dengue serotypes EDIII and JEV EDIII. The sequences of EDIII from DV1 (strain Hawaii), DV2 (NGC strain), DV3 (H87), DV4 (H241), and JEV (T1P1) are shown. Conserved amino acid residues within the epitope region are highlighted by black blocks. Secondary structure elements of DV1 EDIII are shown with arrows (β-strands) and are labeled according to Shresth et al. [29]. Residue numbers correspond to the full-length DV1 E protein. Amino acid residues marked by underlining indicate the sequence of the epitope

Once this linear epitope was identified, the location of epitope (residue 346–360) was shown in the crystal structure of DV1 soluble fragment E (sE) (PDB accession number 3G7T), which is highlighted in green in the crystal structure (Fig. 6a) and in the enlargement of EDIII domain (Fig. 6b). The crystal structure shows that both Thr^346^ and Asp^360^ are surface exposed and accessible in the transition states. Multiple sequence alignment of residues corresponding to this epitope from four serotype DVs and JEV showed that this epitope is distinctive to DV1 with three conserved residues Gly^349^, Arg^350^, Pro^356^(Fig. 6c). The amino acid sequence of the epitope was also identified by BLAST on NCBI and more than 100 E protein peptide belonging to DV1 (data not shown), indicating that this linear epitope is highly conserved among most DV1 strains. Upon further comparison of the five genotypes (type 1 ~ 5) of DV1, only genotype 1 showed one amino acid difference (L351 V) within the epitope.

## Discussion

One primary goal of the present study was to generate neutralizing mAbs that could specifically recognize all DV1 genotype strains. In this study, we generated 12 mAbs against UV-inactivated DV1 and DV1 E395 protein. Among them, 2 mAbs (DV-E1 and DV-E2) specifically reacted to different genotypes of DV1 with no cross-reactivity to the other three DV serotypes and JEV in indirect ELISA. Further characterization of the mAbs with 12 clinical DV strains collected in Taiwan between 1987 and 2006 confirmed the serotype-specificity of both mAbs. The neutralizing activity of both mAbs with DV1 infected C6/36 and Vero cells was evaluated with a semi-quantitative plaque reduction assay. The results showed that DV1-E1 reduced plaque numbers by 57% and 82%, whereas DV1-E2 reduced plaque numbers by 40% and 64% in C6/36 and Vero cells, respectively.

Previous studies showed that the DV serotype-specific neutralizing Abs bind to epitopes located on the EDIII of the recombinant E protein [19, 37–41]. For example, two mAbs that are able to elicit virus-neutralizing activity were located in the 331–352 and 352–368 amino acid regions of DV2 [40]. A similar region, located at amino acids 349–356 of E protein of DV2, was also proposed to be involved in haemagglutination inhibition and virus neutralization in vitro [37]. Furthermore, Thullier et al. identified a mAb that neutralizes DV of all serotypes by binding to the 296–400 segment of the E protein [42]. Recently, a neutralizing human monoclonal antibody (hmAb)-binding epitope of CR4354 also aligned to the 317–327 and 356–364 amino acid regions of E protein segments of DV1, DV2, DV3, and West Nile virus [43]. In this study, we have characterized a neutralizing epitope of DV1-E1 and DV1-E2 by using synthetic peptides spanning EDIII of DV1. Among ten synthetic peptides, P1 - P10, P6 exhibited strong reactivity to both mAbs while the rest of the peptides failed to react with either DV1-E1 or DV1-E2. Trypsin digestion of P6 into two fragments, ^346^TQNGR^350^and ^351^LITANPIVTD^360^, resulted in a loss of reactivity to both mAbs. These results indicate that the putative epitope residues may span across the trypsin cleavage site Arg^350^. Further characterization of epitope recognition residues with truncated peptides derived from P6 showed that elimination of Asp^360^ from P6 completely abolished its ability to be recognized by both mAbs. Alternatively, removal of two and three residues from the *N*-terminus of P6, ND13 and GD12, reduced DV1-E1 activity to 28% and 2% and that of DV1-E2 to 19% and 2% of the original P6 activity. Further shortening of amino acid residues from the *N*-terminus of P6 abolished its affinity to both mAbs. These results suggest the important roles of amino acids T346 and D360 as antibody recognition residues and support TD15 as the minimal requirement for the reactivity of the linearized epitope recognized by both mAbs. Besides, the amino acids Q347 and N348 might also play some functional role in recognition. We found that the epitope displays highly conserved amino acid sequences among different genotypes of DV1 but is diverse from DV2, DV3, DV4 serotypes and other flaviviruses. Notably, there is significant genetic variation and phenotypic difference in virulence among individual strains of specific serotypes and genotypes of DVs. Therefore, the identification of the recognition epitope for both mAbs in the EDIII domain of DV1 cannot exclude the possible existence of other epitopes in distinct regions. Whether binding to different determinants on EDIII influences the mechanism of antibody neutralization of DV remains uncertain.

Binding of antibodies to EDIII domain have been suggested to alter the virus structure, trap the antigen in an already existing conformation, or induce molecular rearrangements prior to epitope binding [32, 44, 45]. EDIII is an immunoglobulin-like module and putatively contains epitopes for neutralizing antibodies and the receptor-binding site [46–48]. Investigating the 3-D structure of the E protein reveals that the Thr^346^ and Asp^360^ residues are indeed spatially exposed on the surface and accessible in the transition states. Asp^360^ of E proteins is conserved in DV1 strains but substituted with Glu, Lys, Tyr and Thr in DV2, DV3, DV4, and JEV, respectively. Similarly, the Thr^346^ of E protein is also conserved in DV1 but substituted with His, Ala, and Lys in DV2, DV3, and DV4, respectively. Furthermore, the Gln^347^ of E protein is also conserved in DV1 but substituted with Val, His, Val and Pro in DV2, DV3, DV4, and JEV, respectively. The carboxylate side chain of Asp^360^ forms a hydrogen bond and a salt bridge to the carboxylate side chain of Glu^362^ and the amino side chain of Lys^363^ with an observed distance of 3.0 and 2.5 Å, respectively. Similarly, the amide side chain of Gln^347^ forms an electrostatic interaction with the guanidinium side chain of Arg^350^ and the pyrrolidine side chain of Pro^370^ with a distance of 3.8 and 3.1 Å, respectively. Furthermore, Gln^347^ of one of the subunits of E protein forms a contact with Thr^163^ of the other subunit of the E protein in the interface. The disruption of the interactions between Asp^360^ and Glu^362^ or between Asp^360^ and Lys^363^ may explain the inhibitory activity of mAbs on DV1 infection.

## Conclusions

Briefly, two anti-E mAbs were generated using native DV1 and a recombinant DV1 E395 protein produced in *E. coli* as immunogens. In addition, identification of mAbs capable of neutralizing DV1 and characterization of the mAb-binding epitopes in the EDIII region, specifically Gln^347^, Asn^348^, and Asp^360^ residues, provide important information for elucidation of virus-mAb interaction and for the development of virus-specific serological diagnostic assay, and may be useful in anti-DV infection therapies and future design of a new DV vaccine.

## Abbreviations

DHF: Dengue hemorrhagic fever
DSS: Dengue shock syndrome
DV: Dengue virus
E: envelope protein
mAb: monoclonal antibody
